# Laser nanostructured gold biosensor for proto-oncogene detection

**DOI:** 10.1038/s41598-023-44372-4

**Published:** 2023-10-11

**Authors:** Cian Hughes, Sithara Sreenilayam, Dermot Brabazon

**Affiliations:** I-Form, Advanced Manufacturing Research Centre, Advanced Processing Technology Research Centre, School of Mechanical and Manufacturing Engineering, Dublin City University, Glasnevin, Dublin-9, Ireland

**Keywords:** Biochemistry, Cancer

## Abstract

The advancement of biosensor research has been a primary driving force in the continuing progress of modern medical science. While traditional nanofabrication methods have long been the foundation of biosensor research, recent years have seen a shift in the field of nanofabrication towards laser-based techniques. Here we report a gold-based biosensor, with a limit of detection (LoD) 3.18 µM, developed using environmentally friendly Laser Ablation Synthesis in Liquid (LASiS) and Confined Atmospheric Pulsed-laser (CAP) deposition techniques for the first time. The sensors were able detect a DNA fragment corresponding to the longest unpaired sequence of the c-Myc gene, indicating their potential for detecting such fragments in the ctDNA signature of various cancers. The LoD of the developed novel biosensor highlights its reliability and sensitivity as an analytical platform. The reproducibility of the sensor was examined via the production and testing of 200 sensors with the same fabrication methodology. This work offers a scalable, and green approach to fabricating viable biosensors capable of detecting clinically relevant oncogenic targets.

## Introduction

The pace of biosensor research has been a significant contributing factor in the quality of modern medical technology. When applied in a clinical setting, they have proven to be a vital tool for the diagnosis, monitoring and treatment of diseases. Traditional research on biosensor fabrication methods has seen a focus on increasing their sensitivity and selectivity^1^. However, as the field has progressed and attomolar^2^ and even zeptomolar^3^ target detection has become possible, we are rapidly approaching a fundamental physical and statistical limit on the sensitivity of sensors available. As a result, in the past decade there has been growing emphasis on decreasing costs and increasing accessibility. The recent global pandemic has further catalysed the interest in low-cost, readily available biosensors as the urgent need for affordable biosensors has become clear not only to the biosensing community^4,5^ but also to the public at large^6^.

Traditionally, nanofabrication makes use of chemical reduction methods which are generally extremely environmentally unfriendly^7^ and rely on batch production. In the field of nanofabrication, the current state-of-the-art in research sees a significant focus on the use of laser-based fabrication methods as a rapid, inexpensive, scalable, and green technology^7–10^. Recent examples of this ongoing laser nanofabrication revolution are ubiquitous in literature and demonstrate the viability, flexibility and scalable nature of these techniques when compared with pre-existing methodologies. As the field of nanofabrication has matured, its progress has impacted and revolutionized a wide variety of other fields. Indeed, laser-based nanofabrication is fast becoming commonplace in metamaterial production, surface design and nanoengineering^11–14^. Similarly, the adoption of modern nanofabrication methods in biosensor research represents a significant shift that underpins much of the recent progress in the field^15^. One example of this is the application of Laser Direct Writing (LDW) to metasurface fabrication^16^. LDW utilises laser ablation to directly deposit nanostructures onto an acceptor substrate by selectively ablating a donor substrate. The nanostructures forming on the acceptor surface condense from the ejected donor material. Interestingly, LDW offers the possibility of more accurate and mass producible metalenses^16^, and metalenses have shown promise as components for improving LDW systems^17^ creating the exciting possibility of a future positive feedback loop, a “Moore’s Law” of nanofabrication. Super-Resolution variants of LDW (SR-LDW) have also been developed, and these have been successfully applied to the fabrication of nanoscale Micro-Electro-Mechanical Systems (MEMS)^18^. The emergence of LDW as a metalens fabrication technique would be unsurprising as similar, less flexible techniques like glancing angle deposition (GLAD) have already been demonstrated to be highly effective for metalens fabrication, allowing for their rapid and inexpensive production^19^.

**Figure 1:**
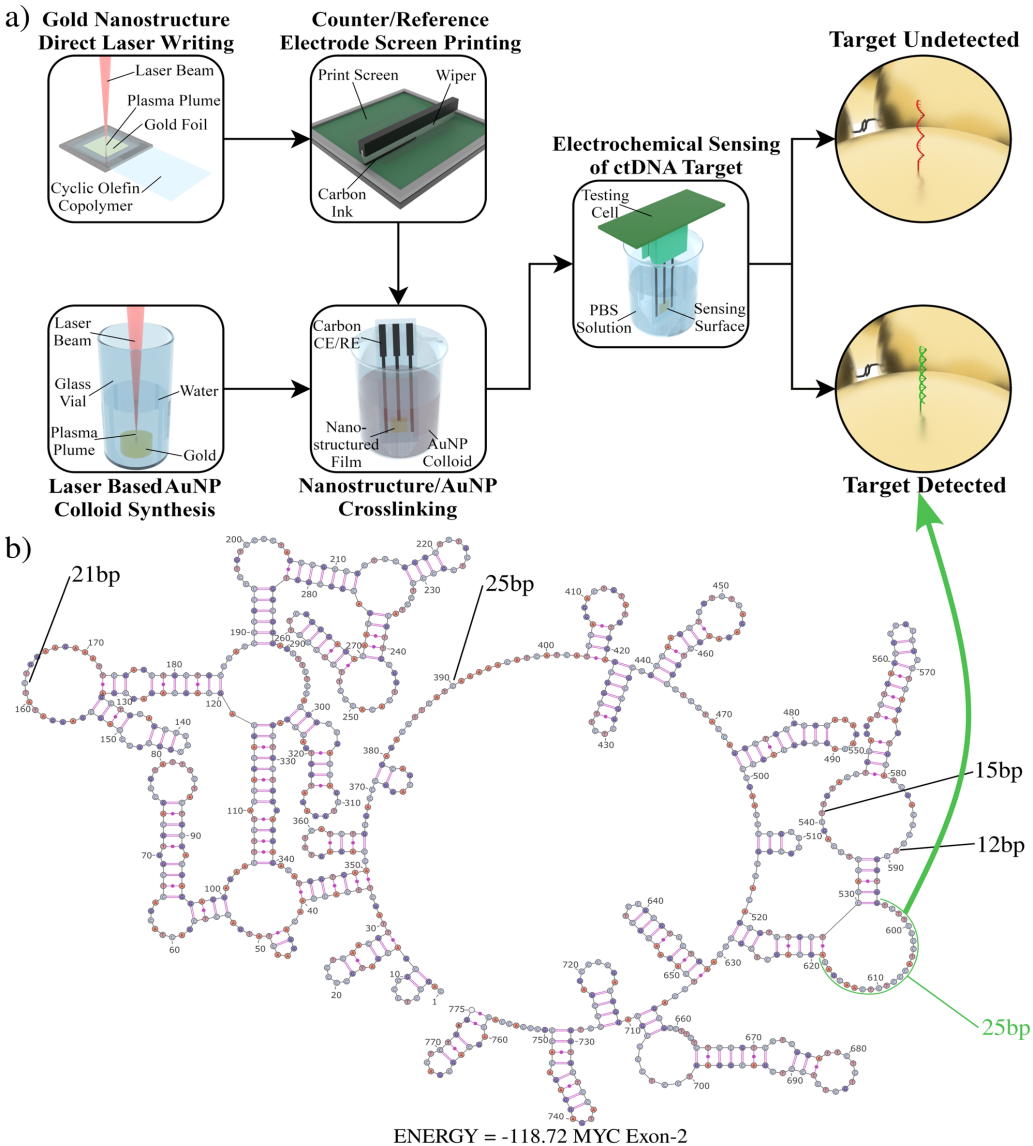
Schematics of proposed gene detection using the developed laser nanostructured gold biosensor. (**a**) Schematic of the sensor fabrication process and (**b**) the single strand secondary structure of exon 2 of the c-MYC gene with the target sequence selected highlighted in green. If the target sequence is present in the sample being tested, it binds to the probes on the sensor surface triggering a change in its electrochemical properties. The single strand secondary structure of exon 2 of the c-MYC was plotted using mFold (version 3.6), that structure was converted to a diagram using SnapGene (version 6. 1. 1), and the picture was then colourised/annotated for clarity using InkScape (version 1.2.2).

In the realm of sensor fabrication, non-LDW techniques have already shown great promise for chemical sensor fabrication. For example, the technique of Highly Regular Laser-Induced Periodic Surface Structuration (HR-LIPSS) has been applied to the production of highly sensitive heavy-metal sensor platforms^20^. Here, inspired by such successes of laser nanofabrication we leverage several emerging methods (building upon our previous work^21^) to prepare a nanostructured biosensor, aiming to confer the advantages of laser-based fabrication onto biosensor fabrication, advancing the field towards the goals of producing inexpensive biosensors in abundance. The methods used were continuous flow Laser Ablation Synthesis in Solution (LASiS)^22,23^ and Confined Atmospheric Pulsed-laser deposition (CAP)^24,25^. A schematic of sensor fabrication process is shown in Fig. 1. LASiS is a technique wherein a donor material is ablated in liquid, resulting in the precipitation of nanoparticles in a colloidal suspension. The other technique, CAP, is a type of reverse transfer LDW technique wherein the ablation plume ejected from the donor material is confined, extending the lifetime of the plasma, and facilitating deposition without the need for the ablation to be carried out in a vacuum. To demonstrate the use of these laser-based nanofabrication methods to fabricate a functioning biosensor platform, a test-case was devised using the system developed for the detection of a proto-oncogene sequence of diagnostic significance.

The gene known as MYC proto-oncogene, bHLH transcription factor (commonly referred to simply as c-Myc) is part of the basic Helix-Loop-Helix-leucine Zipper (bHLHZip) family of regulator genes, a family responsible for regulating the expression of other genes that play key roles in the cell growth cycle^26^ and metabolic activity^27^. Within the bHLHZip family, the MYC subfamily of genes code for proteins that heterodimerize with another protein called MYC associated factor X (MAX) as part of the MYC/MAX/MXD network^28^. Once MYC/MAX dimers have formed they bind to E-Box DNA sequences, activating a plethora of genes responsible for cell growth, differentiation, and DNA metabolism^29^, among other functions. As might be expected based on this understanding of their pro-proliferative activity, many members of the MYC family of proteins are known oncogenes with c-Myc being the most significant of these^30^. The c-Myc oncogene is found to be overexpressed in most human cancers^31^, playing a primary role in tumourigenesis and tumour maintenance^32^, along with an important but less significant role in tumour metastasis^33^. The level of c-Myc present in malignant cells has also been strongly associated with more aggressive cancer and poorer prognoses for patients^34^. As such, c-Myc presents an ideal target for the development of a cancer biosensor through cell free DNA (cfDNA) analysis.

Cancer patients are generally found to have significantly higher levels of cfDNA than healthy patients, with the excess cfDNA present resulting from oncometabolism being referred to as circulating tumor DNA (ctDNA). In the past, the relatively low concentrations of cfDNA (a mean of 12.54 ng/mL for healthy patients and up to 79.51 ng/mL for cancer patients^35^) have hindered their study, but recent advances in DNA amplification have begun to facilitate the study of these biomarkers identifying them as a promising candidate for future diagnostic applications. Through the amplification of cfDNA, the quantitative study of ctDNA with relatively low sensitivity biosensors has become possible, significantly lowering the barrier-to-entry for researchers. Of the limited number of common ctDNA fragments studied c-Myc is one of those most commonly found to have diagnostic significance^36–38^.

To detect c-Myc fragments, a short 25 nucleotide long thiolated probe was used. This probe was selected based on an examination of the ssDNA secondary structure of the c-Myc sequence. The probe selected had the following sequence:

5′-TGTCGTTGAGAGGGTAGGGGAAGAC-3′-SH

This sequence is an antisense strand corresponding to nucleotides 595–619 of exon 2 of the c-Myc gene. This target section of the c-Myc gene has the following sequence:

5′-GTCTTCCCCTACCCTCTCAACGACA-3′

Based on mfold software secondary structure predictions^39^, this region of exon 2 is expected to form the longest unpaired loop in the secondary structure of the gene (Fig. 1b), making it an ideal candidate for a diagnostically useful target subsequence of c-Myc ctDNA. In previous work we have demonstrated that modern, LDW based nanofabrication techniques can be used to produce an electrode capable of responding to surface interactions^21^. Herein, we build upon this groundwork by demonstrating the first use of a newly developed electrochemical biosensor platform fabricated via laser-based methods in the detection of an oncogenic ctDNA fragment of clinical interest. This sensor can be fabricated at low-cost and large-scale highlighting a potentially promising path towards the goal of creating affordable, readily available nanoengineered biosensors.

## Results and discussion

### Large scale production of biosensors

To demonstrate the scalability of the developed fabrication method, we performed an experiment producing the sensors at large scale. The experiment resulted in the fabrication of over 200 sensors in a single day (Fig. S1). With each step of the fabrication process being readily automatable, a significant increase in this already high production rate could be achieved with further development. Moreover, automating the process could improve the accuracy and reliability of the sensors by reducing variance in their quality. Notably, the human errors observed during manual production (such as poor alignment of components and inconsistent ink thickness during screen-printing) would be minimized by automation, thus significantly improving the quality and homogeneity of the sensors produced. These improvements in scalability and quality would be expected to have a highly beneficial impact on the practical applications of the fabricated sensors.

### Electrochemical monitoring of fabrication process

**Figure 2:**
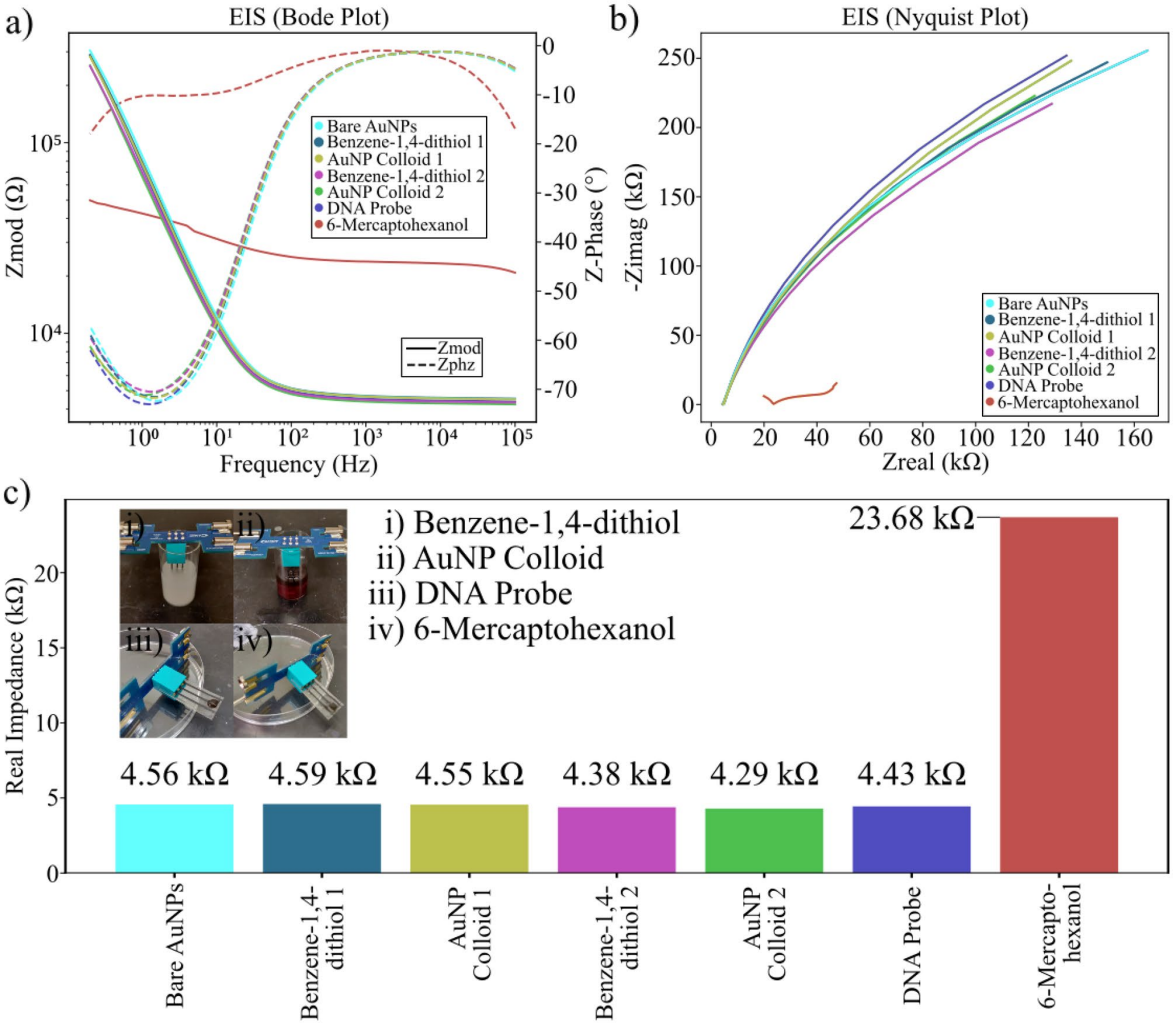
Process steps with sample examination. (**a**) Bode and (**b**) the Nyquist spectra for the sample examined at each step of the fabrication process; (**c**) Real impedances of the samples tested at each stage of the fabrication process (inset photographs from the process steps).

During production, a sampling of the sensors produced were subjected to Electrochemical Impedance Spectroscopy (EIS) testing between every step of the fabrication process. These sensors were not used during electrochemical response testing to ensure that repeated EIS testing did not degrade the surfaces of those sensors used in that experiment. Based on this data, a comparison was made between the properties of the sensor surface at the various steps of production to understand the fabrication process (Fig. 2, Figs. S2–S8). It was noted that the electrochemical property of the sensor surfaces as measured by EIS did not appear to vary significantly during the various thiol submersions, colloid submersions, or introduction of the DNA probe. However, there is a clear and large shift in the EIS spectrum in response to the passivation step of the fabrication process, wherein the surface was functionalised using 6-mercaptohexanol. To quantify this, the Nyquist plots (Fig. 2b) were used to determine the real impedance of each sample tested. The variation of this real impedance during fabrication was then examined more closely (Fig. 2c, Table S1).

During the passivation step of fabrication, there is a clear fivefold increase in the real impedance of the sensing surface (Fig. 2c). Based on an examination of the literature it is entirely expected that the passivation of the electrode should increase its impedance^40^. The observation of this effect in the EIS spectrum during this experiment is indicative of a signal denoting a surface interaction in the sensor, which is key to the design of a viable electrochemical biosensor. In addition, the size of this increase is known to be proportional to the thickness and density of the passivation layer. Based on this, the passivation impedance observed could be used in future experiments to optimise the passivation step of fabrication to maximise sensor performance. This passivated impedance could also be useful as a quality assurance test in the future, allowing us to verify that sensors are functioning correctly and have the desired surface properties before declaring them fit for testing.

### Electrochemical response of sensor to c-Myc fragment detection

**Figure 3:**
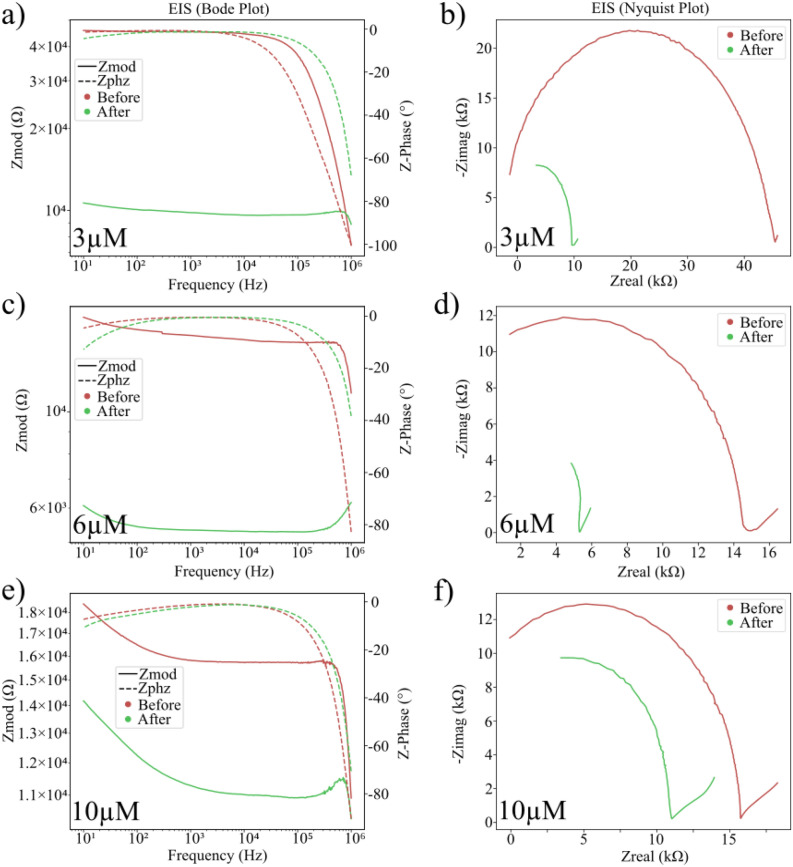
EIS plots of the target c-Myc fragment, before (red) and after (green) exposure. The plots shown include (**a**) the Bode plot at a concentration of 3 µM, (**b**) the Nyquist plot at a concentration of 3 µM, (**c**) the Bode plot at a concentration of 6 µM, (**d**) the Nyquist plot at a concentration of 6 µM, (**e**) the Bode plot at a concentration of 10 µM, and (**f**) the Nyquist plot at a concentration of 10 µM.

Following the exposure of the sensors of c-Myc target fragments, a significant decrease in real impedance was observed in every sample. This decrease in real impedance before and after exposure is clearly visible in every case in both the Bode and Nyquist plots of the impedance spectra (Fig. 3). In addition, comparing these plots for differing concentrations shows a clear trend that the gap between the real impedance before and after exposure to the target analyte is larger for smaller concentrations. This implies a correlation between this change in impedance and the concentration of the target analyte.

The semicircular feature on the Nyquist plots suggests the fabricated sensors are behaving as diffusive surfaces^41^. However, upon attempts to perform equivalent circuit modelling (ECM) it was found that the spectra did not fit the expected behaviour of the diffusive Randles’ circuit commonly used to model simple diffusive surface electrodes. Further experimentation found the data to be most closely fitted by a model consisting of 3 diffusive Randles’ circuits in parallel, resulting in an extremely short, diffusive transmission line model (Fig. S9). Interestingly, this 3-element transmission line model suggests the sensors produced feature 3 significantly distinct diffusive electrode regimes, despite being comprised of only 2 distinct nanostructured components (the CAP deposited nanostructures and the LASiS fabricated crosslinking nanoparticles). It could be speculated that the third diffusive element may be a reading from a non-sensing element of the sensor, and therefore future work to discern its source could yield further improvements to the sensor design.

Analysis of the EIS data obtained (Table 1, Table S1) using the equivalent circuit model fitted followed by ANOVA analysis of the various responses observed yielded two statistically significant linear regressions relating response to analyte concentration (Table 2, Tables S3–S4).

**Table 1:** Variation of dR_Total_ and %dZ_Real_ as a result of target analyte concentration.

Concentration (µM)	dR_Total_ (Ω)		%dZ_Real_ (%)	
3	− 2.01E + 05	− 2.24E + 05	− 68.143	− 78.790
4	− 1.58E + 05	− 2.17E + 05	− 74.801	− 75.681
6	− 9.24E + 04	− 4.06E + 04	− 67.448	− 64.298
8	− 1.71E + 04	− 1.24E + 05	− 58.870	− 52.011
10	1.09E + 05	2.55E + 04	− 5.439	− 29.905

**Table 2:** Summary of ANOVA results for the response equations derived.

Response	R^2^	Adjusted R^2^	Adequate precision	p
dR_Total_	0.8602	0.8427	13.602	0.0001
%dZ_Real_	0.7667	0.7376	10.847	0.0009

It was found that the response of the total resistivity of the ECM resistor component in the sensing surfaces (dR_Total_) was related to the micromolar concentration of target analyte (C_M_) by the following equation:

$${C}_{M}=\frac{{dR}_{Total}+3.44 {e}^{5}}{40390}$$

Similarly, it was found that the percentage change in the real impedance of the sensing surfaces (%dZ_Real_) was related to the micromolar concentration of target analyte by the following equation:

$${C}_{M}=\frac{{\%dZ}_{Real}+107.19}{8.70}$$

**Figure 4:**
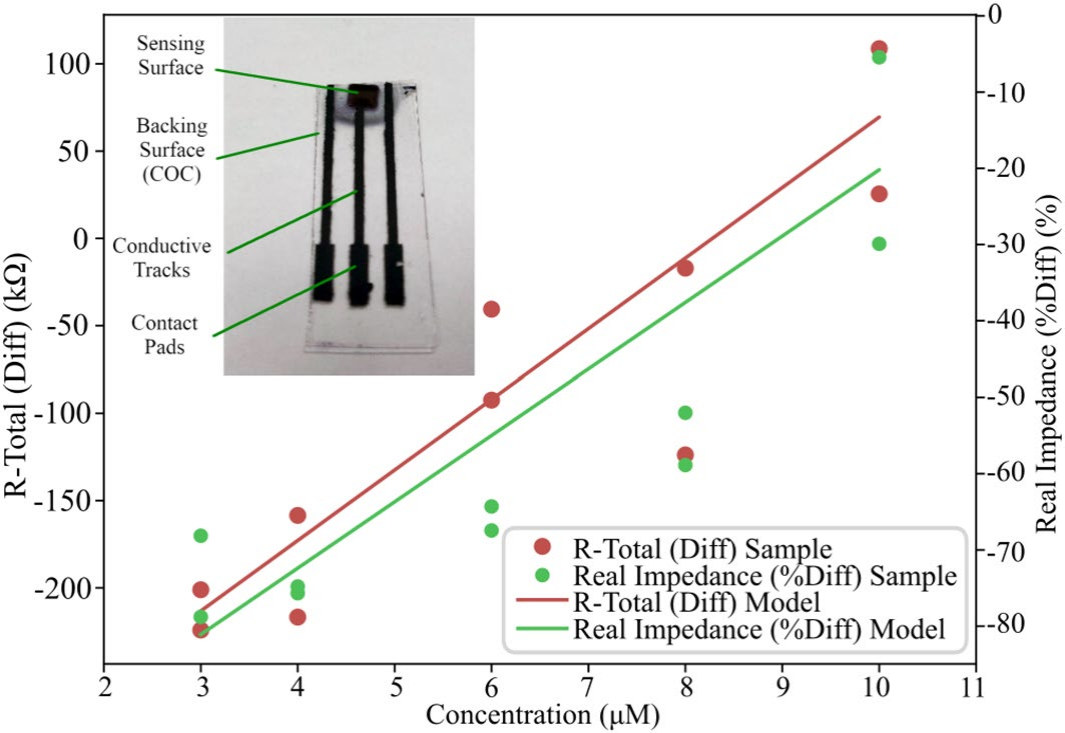
Plots of the models for (**a**) concentration versus dR_Total_ and (**b**) concentration versus %dZ_Real._ Inset: developed biosensor.

These response equations describe a quantitative response in the sensing surfaces as a function of the amount of target analyte present. They both agree with each other, outlining positive and linear relationships between the resistance and impedance of the sensor and the concentration of target analyte present (Fig. 4).

To evaluate the relative performance of each of equations for analytical testing, the limit of detection (LoD) for each measurement was calculated according to the formula LoD = 3σ/slope. Here σ is the standard deviation. Using this formula, the LoD for the dR_Total_ regression and %dZ_Real_ regression were found to be 3.26 µM and 3.18 µM, respectively. Based on this both regressions offer similar sensitivity under the testing conditions.

## Conclusions

Demonstrated here, a viable biosensing platform has been successfully fabricated leveraging emerging, novel, laser-based nanofabrication techniques. In contrast with traditional methods, these new fabrication techniques make possible the production of these sensors in fast, low-cost, scalable, and green manner. Reasonably large-scale production of biosensors via the method outlined was successfully achieved, yielding hundreds of sensors per day. Based on the current fabrication method, the material cost is estimated to be $4.75 per sensor (Table S5) at current market prices for the materials used. The fabrication method is readily automatable, making it possible to significantly increase the scale of production and thereby further reduce the cost per sensor.

In this work, the fabrication method allowed for the production of sensors at a rate of approximately 2 per minute. This process yielded sensors with an LOD of between 3.18 and 3.26 µM, demonstrating their ability to reliably detect the target analyte in the conditions tested. In addition, 2 statistically significant regression equations were found (p = 0.0001 and p = 0.0009) demonstrating the quantitative capability of the sensors. These equations showed the same relationship trend, both describing a positive correlation between the resistance of the sensing element and the concentration of target analyte present in the testing solution. Based on this accumulated evidence, we conclude that we have managed to successfully produce a viable biosensor using the novel fabrication methods developed. This fabrication method, of course, still offers room for future development and improvement suggesting an interesting potential path to our future goals of inexpensive and mass producible diagnostics. Despite their demonstrable detection capability and quantitative capability, the sensitivity of these sensors is currently not enough for use in a clinical setting. However, there is much room for further optimisation of the design without sacrificing the benefits conferred by using novel fabrication techniques. Future optimisation of untested parameters such as feature size, particle size, passivation layer thickness/density, and surface loading offer paths forward for development of a clinically viable biosensor. In addition, the automation of this fabrication technique in the future will reduce the impact of human error and inaccuracy on the fabrication process. This would further increase the accuracy of the sensors produced. Experimental characterisation has already identified quantifiable properties such as passivation impedance that offer quick, easily obtainable metrics for quality assurance in the fabrication process. Through the introduction of more accurate, reproducible automated methods and proper quality assurance an optimised sensor fabricated via this method would be expected to have significantly improved sensitivity, accuracy, and precision over the current proof-of-concept design presented herein.

These results demonstrate the potential of new laser-based nanofabrication techniques as a biosensor fabrication method. Currently, they are capable of producing functional, sensitive electrochemical biosensors capable of detecting a diagnostically relevant ctDNA fragment of the c-Myc exon 2 proto-oncogene. However, it should be acknowledged that their sensitivity is not yet high enough for reliable use in an applied, clinical diagnostic setting at this early stage and there are many potential paths forward to bridging this gap in capabilities.

Future work to expand upon this will include finely controlled optimisation of the sensor platform, automation of the production process, the development of quality assurance techniques to maximise the reliability of the final product, and the use of the platform for the detection of other diagnostically relevant targets in biological matrices. With these continued future developments, we believe that this fabrication methodology may yield a pathway to inexpensive, green, and clinically applicable biosensors.

## Methods

### Fabrication of the biosensor platform

The biosensor was fabricated via a combination of the CAP and LASiS processing methods to produce a nanostructured matrix suitable for use as a sensor transducer layer. Other, simpler elements of the sensor such as contacts, counter electrode, and reference electrodes were produced using screen- printing technique. Overall, the fabrication process was comprised of three consecutive steps, and these steps were as follows: (1) CAP deposition of nanostructures on a polymer base; (2) Single-step screen-printing of contacts, counter electrodes, and reference electrodes; and (3) Surface nanostructure crosslinking to produce final biosensor matrix. This process results in the production of a small, flexible, nanostructured gold surface with high conductivity, high surface area, and contacts suitable for use in common electrochemical testing apparatus.

### CAP deposition of nanostructured gold electrodes

CAP deposition is an atmospheric, reverse transfer LDW technique wherein plasma confinement effects are exploited to enhance deposition and avoid the need for a vacuum to enable nanostructure deposition. A 99.9% gold target (10 mm × 10 mm × 0.188 mm) was prepared from a sputtering target (Agar Scientific, UK) and used as a donor substrate to deposit a 5 mm × 5 mm square nanostructured gold surface on a Cyclic Olefin Copolymer (COC) acceptor substrate sheet (ZeonorFilm ZF14-188, Zeon Chemical L.P. Japan) via the CAP deposition method^24^. The CAP process was performed using a 1064 nm Nd:YAG laser at the parameters listed in Table S6.

### Screen-printing of contacts, counter, and reference electrodes

Screen-printing (using a Dek 248 semi-automatic printer) was used to print the shape of contacts (Fig. S10), a counter electrode, and a reference electrode in an inert conductive carbon ink (Ercon E3178, Ercon Ink, USA). Following printing, the samples were left to dry at room temperature in atmospheric conditions for an hour yielding solid, well adhered, contiguous, and conductive contacts for the sensing surfaces. This printing process was performed using a screen allowing for the production of up to 50 sensors at once.

### LASiS production of nanoparticle colloid

A continuous flow LASiS production system^22^ was used to produce a gold nanoparticle colloid for nanostructure crosslinking. To produce the colloid, a gold target was ablated into suspension in deionised water with the LASiS parameters outlined in Table S7. This process yielded an aqueous gold nanoparticle colloid with a mean diameter of 14.08 nm with a standard deviation of 3.02 nm. These measurements were performed using a Microtrac NANO-Flex 180° dynamic light scattering machine.

### Surface nanostructure crosslinking for enhanced electrical conductivity

Following screen-printing, the sensors were then given their final desired properties by the crosslinking of non-contiguous adjacent nanostructures. This step enhanced the active surface area of the sensors in addition to their conductivity. To perform this fabrication step, the sensor surfaces were first submerged for 5 min in 100 mL of a 0.1% (w/v) solution of benzene-1,4-dithiol (99% GC, Sigma-Aldrich, US) in a 50:50 water:ethanol solvent. They were then submerged in 100 mL of aqueous gold nanoparticle colloid for 5 min. This process was then repeated, submerging for a further 5 min in benzene-1,4-dithiol, and 5 min in gold nanoparticle colloid to complete the crosslinking step. Between each submersion, samples were rinsed with ethanol to minimise cross-contamination between reagent containers.

### Sensor surface functionalisation and passivation

Each sensing surface was treated with 100 µL of 10 µM solution of thiolated DNA probe (supplied by Integrated DNA Technologies, Inc., USA) in phosphate buffered saline (PBS) solution for 15 min. Following this, each sensing surface was treated with 100 µL of 5% (v/v) 6-mercaptohexanol (97%, supplied by Sigma-Aldrich, USA) in a 50:50 water:ethanol solvent for 30 min.

### Electrochemical determination of sensor response curve

To establish a baseline against which to compare, each sensor was first examined by EIS (Gamry Interface 1000E). A dilution series of strands of the target DNA segment (Integrated DNA Technologies, Inc., USA) in PBS solution was prepared, yielding 10 µM, 8 µM, 6 µM, 4 µM and 3 µM solutions. For each concentration, 2 sensors were then each exposed to 100 µL of analyte solution for 30 min. Following exposure, the sensors were rinsed with a small amount of ethanol to remove unbound target DNA before being submerged in a PBS solution for testing. The tests were performed in a 3-electrode configuration using a standard testing cell (Fig. S11, supplied by Gamry Instruments Inc) connected to the potentiometer via common banana connector cables. All EIS measurements herein were carried out according to the parameters in Table S8.

### Supplementary Information

Supplementary Information.

## Data Availability

The datasets used and/or analysed during the current study available from the corresponding author on reasonable request.
