# Supplementary material for: Laser nanostructured gold biosensor for proto-oncogene detection
Source: Sci Rep. 2023 Oct 11;13:17196. doi: 10.1038/s41598-023-44372-4 (PMC10567688; doi:10.1038/s41598-023-44372-4)
Supplement: Supplementary file 1 — Supplementary Information. [file 41598_2023_44372_MOESM1_ESM.docx]

Supporting Information

Laser Nanostructured Gold Biosensor for Proto-oncogene Detection

Cian Hughes^1^, Sithara Sreenilayam^1^, Dermot Brabazon^1*^

^1^I-Form, Advanced Manufacturing Research Centre, & Advanced Processing Technology Research Centre, School of Mechanical and Manufacturing Engineering, Dublin City University, Glasnevin, Dublin-9, Ireland

Corresponding Author E-mail: dermot.brabazon@dcu.ie


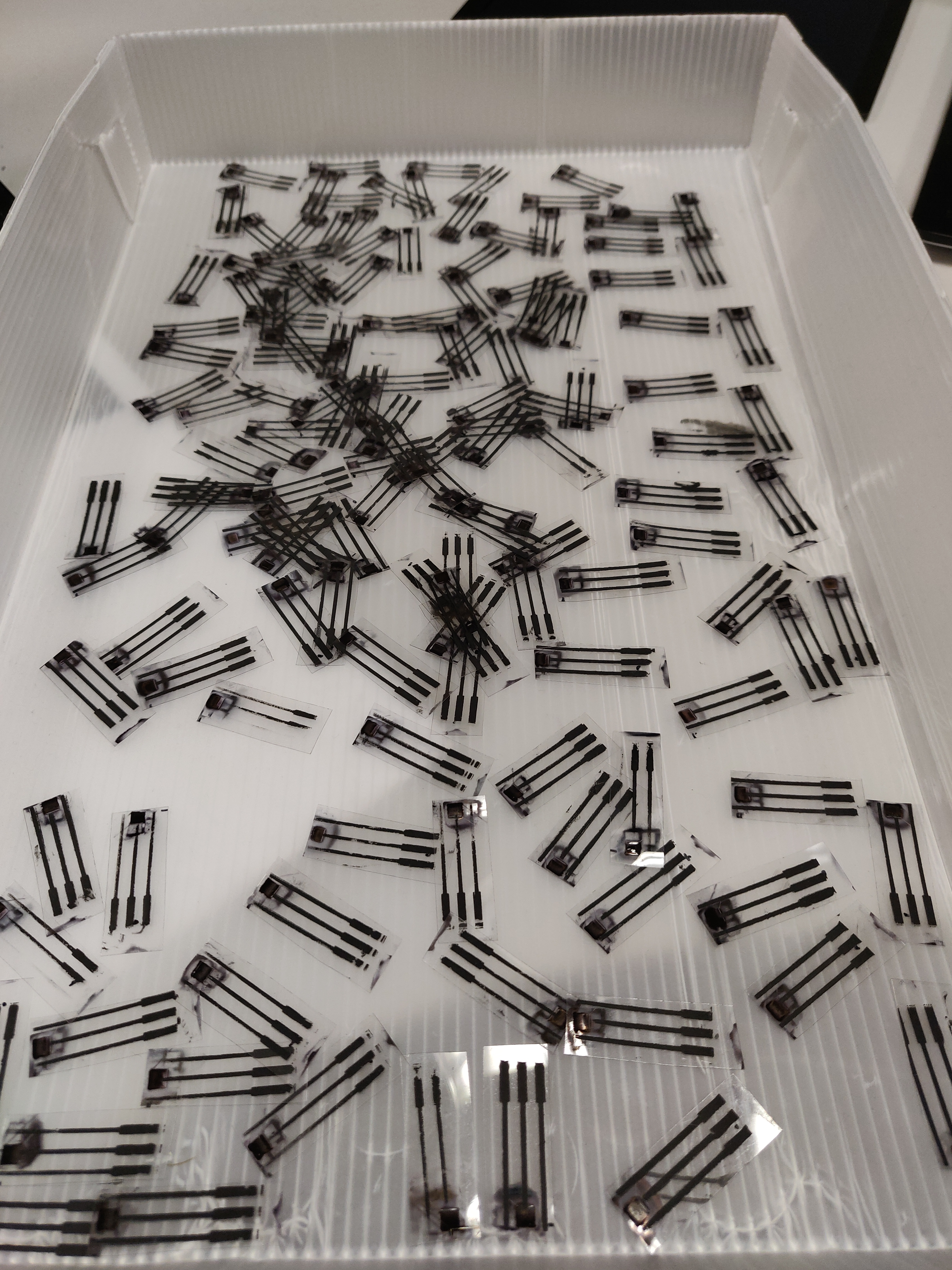
Fig. S1. A photograph of a batch of unfunctionalized sensors produced during this study.


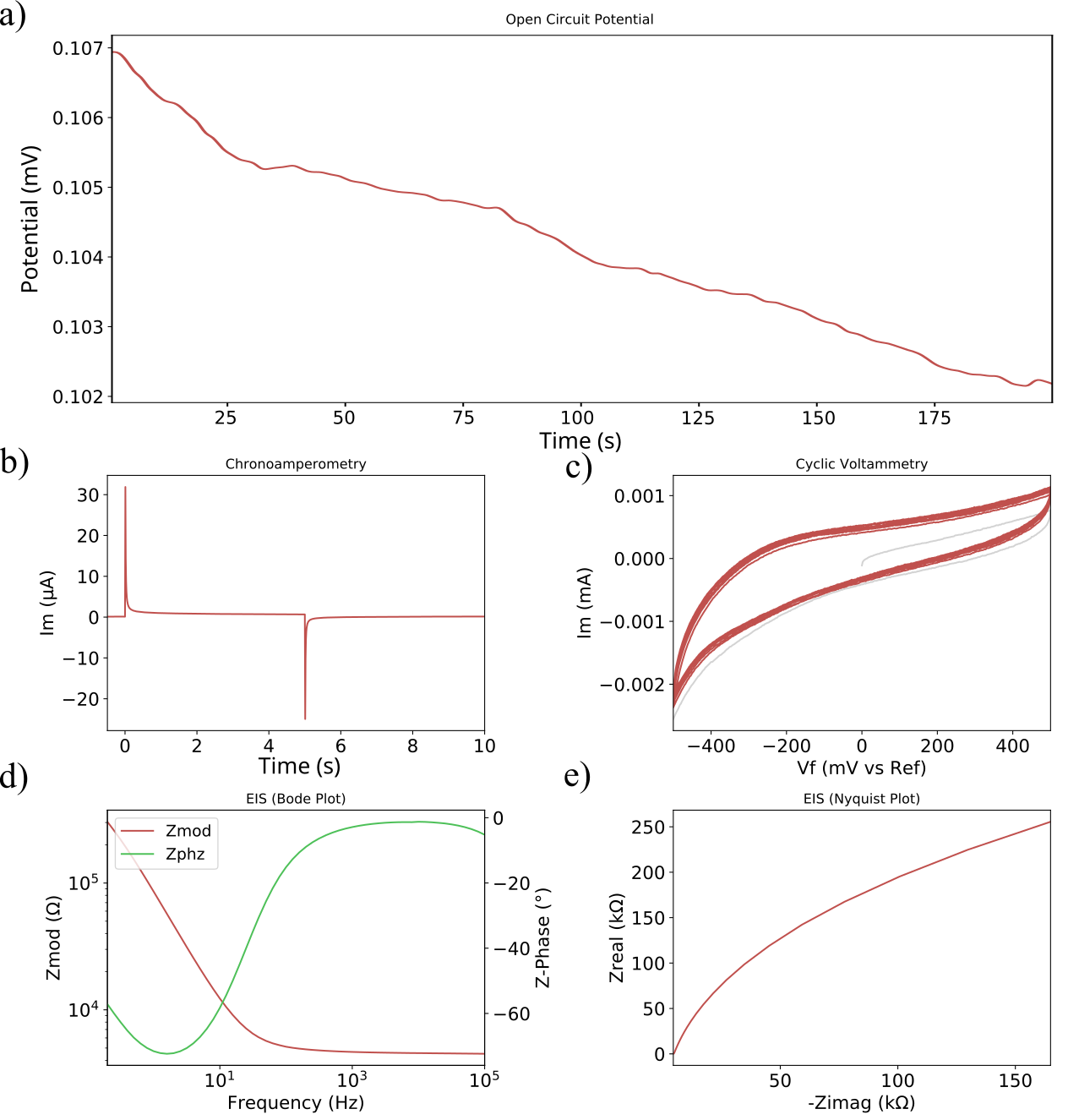
Fig. S2. Conductometric data for the unfunctionalised gold nanostructured surface (Bare AuNPs). This data includes a the open circuit potential, b a chronoamperometry scan, c a cyclic voltammogram, and an electrochemical impedance spectrum represented as d a Bode plot and e a Nyquist plot.


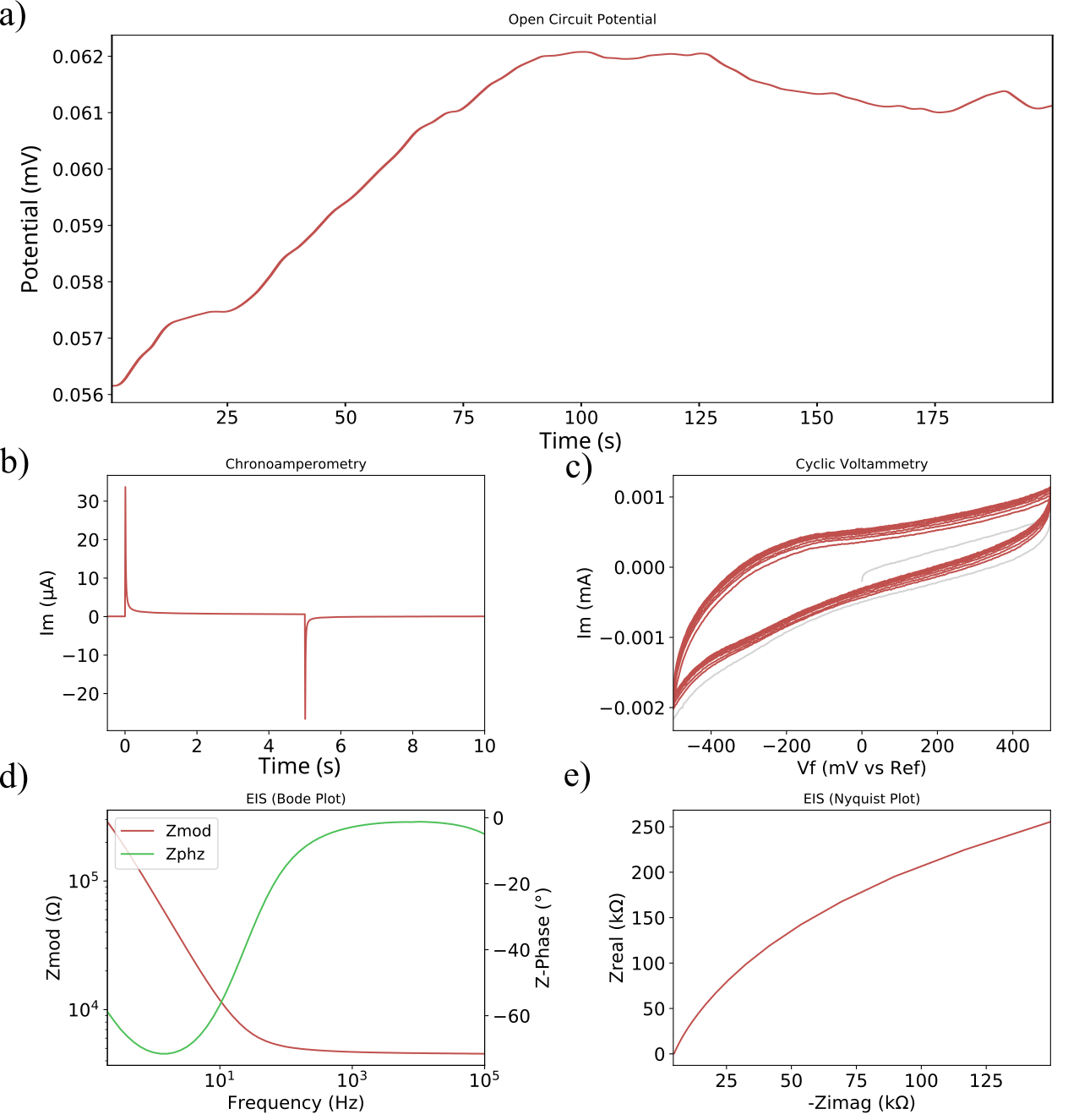
Fig. S3. Conductometric data for the sensor surface following its first dithiol exposure (Benzene-1,4-dithiol 1). This data includes a the open circuit potential, b a chronoamperometry scan, c a cyclic voltammogram, and an electrochemical impedance spectrum represented as d a Bode plot and e a Nyquist plot.


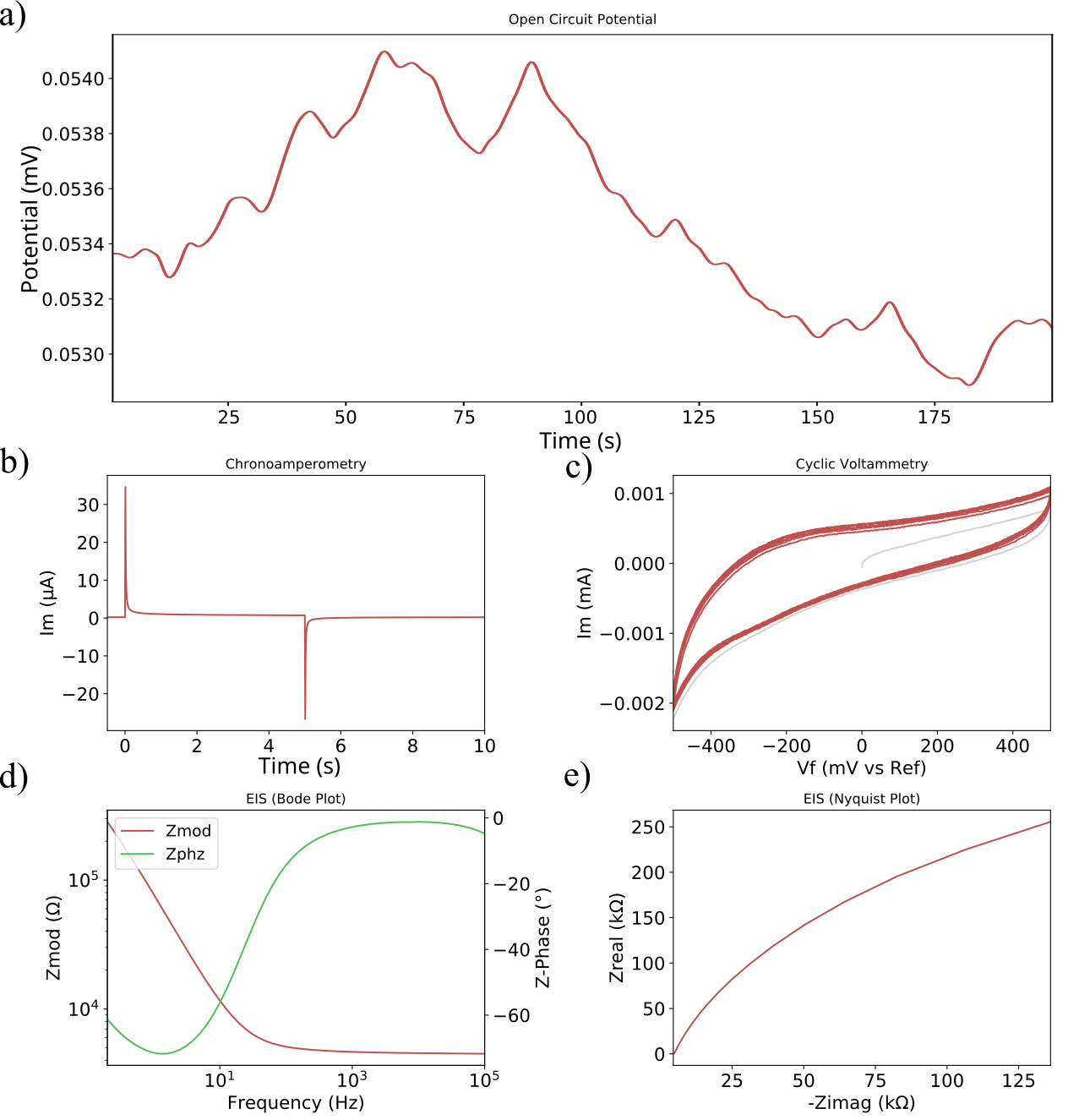
Fig. S4. Conductometric data for the sensor surface following its first colloid exposure (AuNP Colloid 1). This data includes a the open circuit potential, b a chronoamperometry scan, c a cyclic voltammogram, and an electrochemical impedance spectrum represented as d a Bode plot and e a Nyquist plot.


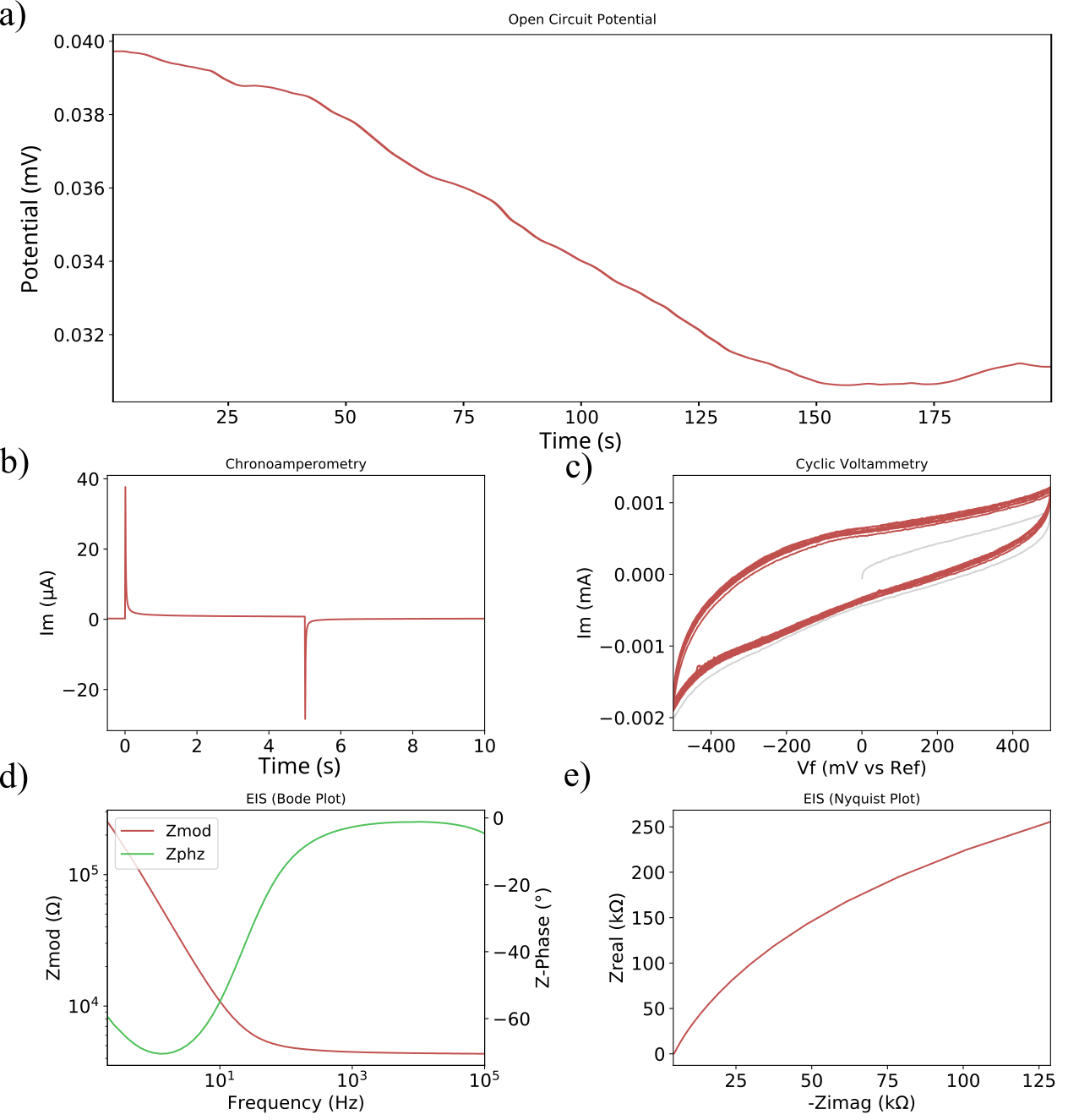
Figure S5. Conductometric data for the sensor surface following its second dithiol exposure (Benzene-1,4-dithiol 2). This data includes a the open circuit potential, b a chronoamperometry scan, c a cyclic voltammogram, and an electrochemical impedance spectrum represented as d a Bode plot and e a Nyquist plot.


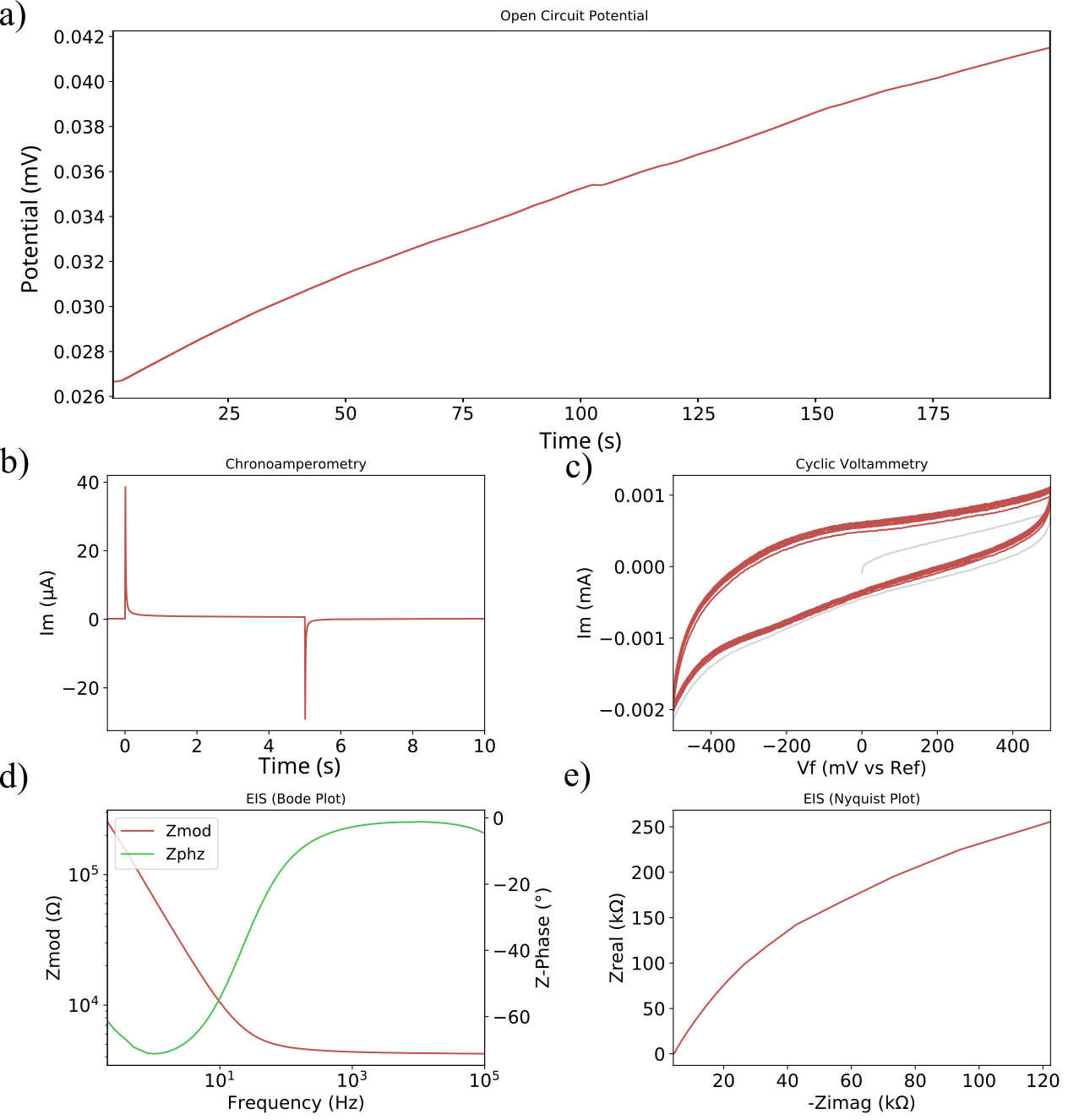
Fig. S6. Conductometric data for the sensor surface following its second colloid exposure (AuNP Colloid 2). This data includes a the open circuit potential, b a chronoamperometry scan, c a cyclic voltammogram, and an electrochemical impedance spectrum represented as d a Bode plot and e a Nyquist plot.


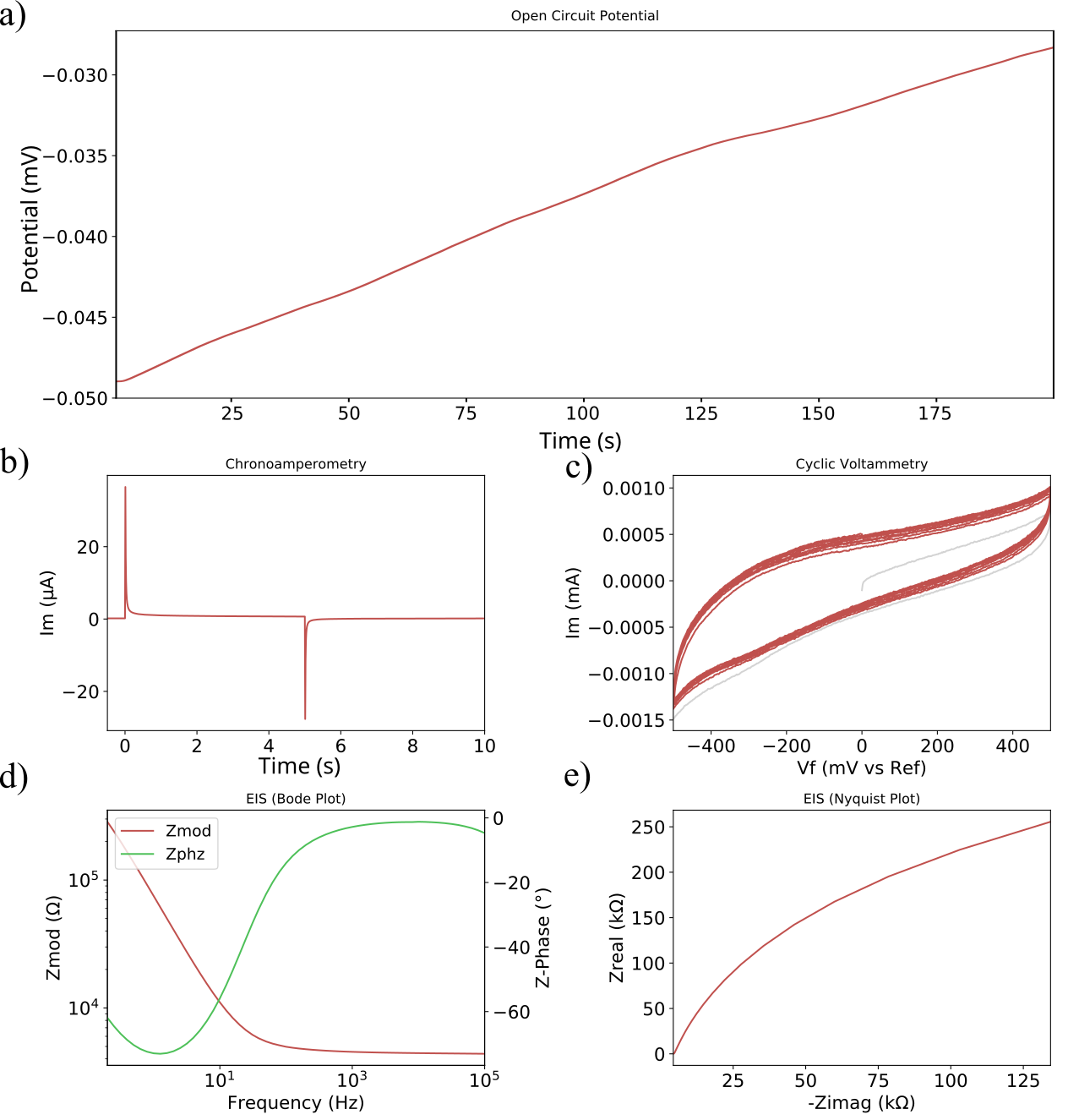
Fig. S7. Conductometric data for the sensor surface following its exposure to the detector probe (DNA Probe). This data includes (a) the open circuit potential, (b) a chronoamperometry scan, (c) a cyclic voltammogram, and an electrochemical impedance spectrum represented as (d) a Bode plot and (e) a Nyquist plot.


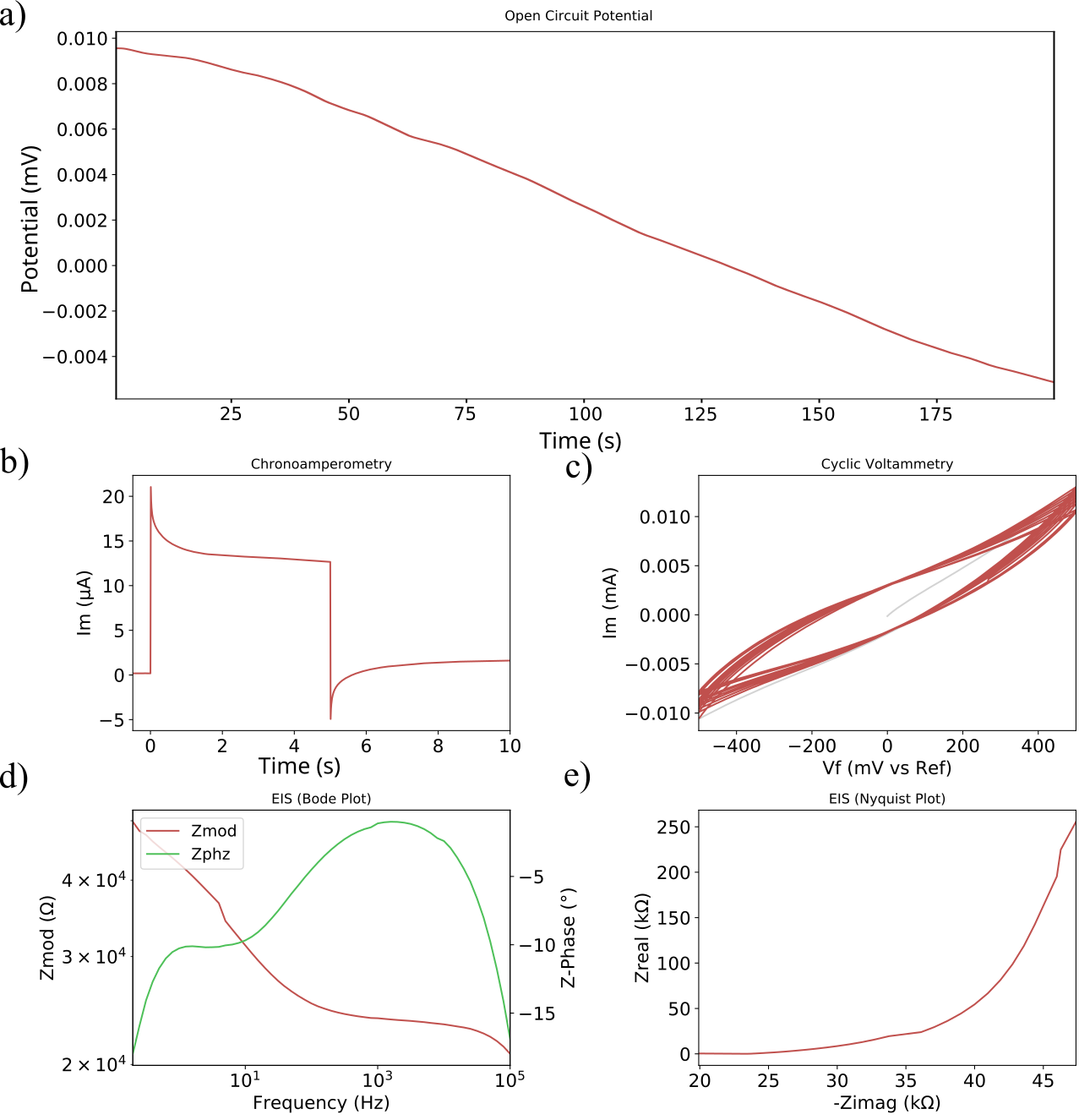
Fig. S8. Conductometric data for the sensor surface following its exposure to a passivating agent (6-Mercaptohexanol). This data includes a the open circuit potential, b a chronoamperometry scan, c a cyclic voltammogram, and an electrochemical impedance spectrum represented as d a Bode plot and e a Nyquist plot.

Table S1: The real impedance as measured by EIS following each step of the fabrication process.

| **Fabrication Step** | **Real Impedance (kΩ)** |
| --- | --- |
| Bare AuNPs | 4.56 |
| Benzene-1,4-dithiol 1 | 4.59 |
| AuNP Colloid 1 | 4.55 |
| Benzene-1,4-dithiol 2 | 4.38 |
| AuNP Colloid 2 | 4.29 |
| DNA Probe | 4.43 |
| 6-Mercaptohexanol | 23.68 |


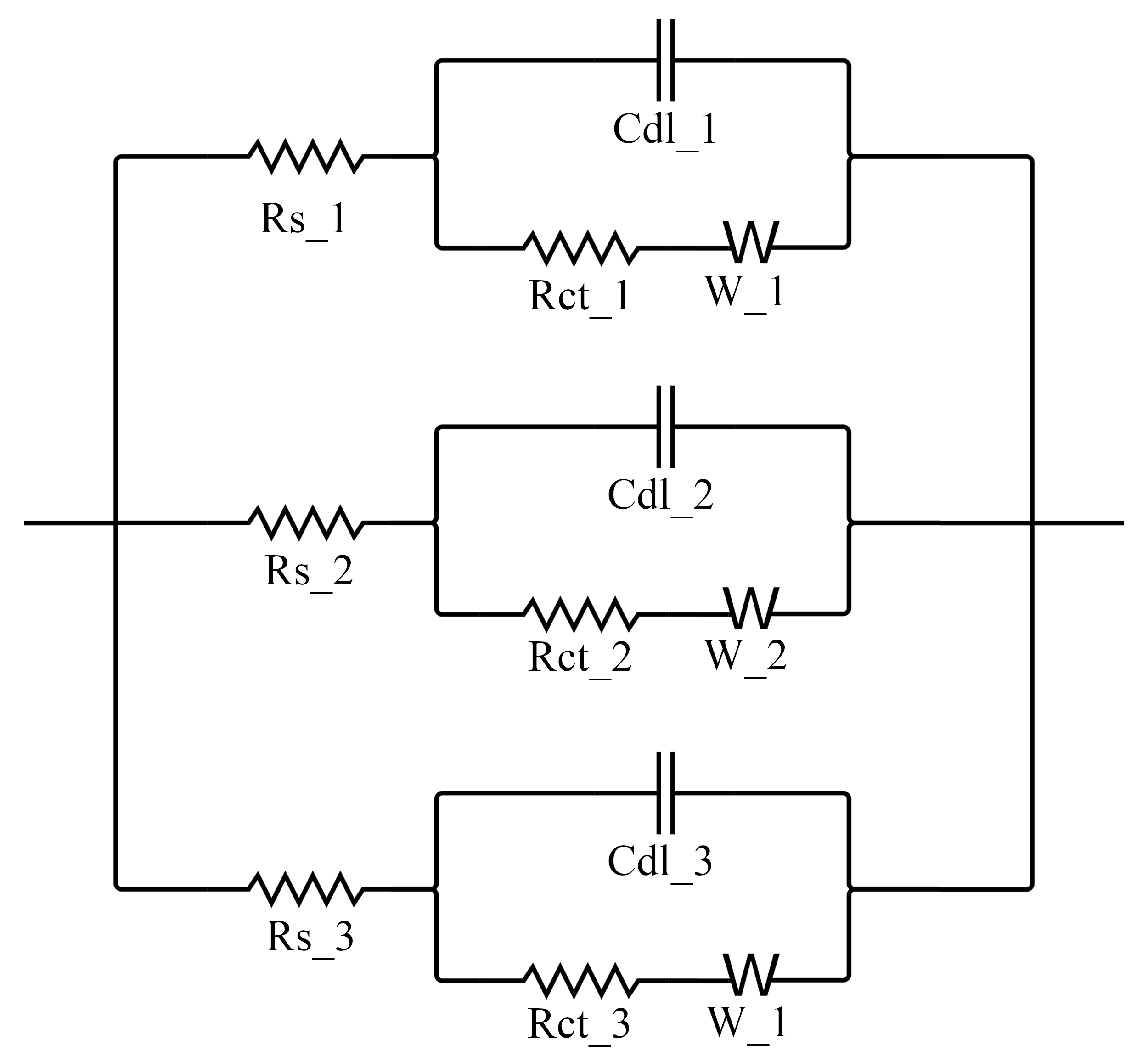
Fig. S9. A schematic of the n=3 transmission-line model derived, with each component labelled.

Table S2: Each measured response of the sensor to varying concentrations of target analyte, as calculated via EIS equivalent circuit modelling.

| Sample | 1 | | 2 | 3 | 4 | 5 | 6 | 7 | 8 | | 9 | 10 |
| --- | --- | --- | --- | --- | --- | --- | --- | --- | --- | --- | --- | --- |
| Conc (µm) | 3 | 4 | | 6 | 8 | 10 | 3 | 4 | 6 | 8 | | 10 |
| Rs_1 (after) (Ω) | 5.39E+03 | 1.01E+04 | | 3.62E+03 | 2.84E+04 | 2.07E+04 | 1.56E+04 | 9.88E+03 | 1.14E-04 | 1.34E+04 | | 3.14E+04 |
| Rct_1 (after) (Ω) | 5.88E-02 | 3.81E+03 | | 3.64E+02 | 7.04E+02 | 6.51E+04 | 1.41E+04 | 3.81E+00 | 7.22E-01 | 2.37E+03 | | 1.33E+04 |
| Cdl_1 (after) (F) | 1.23E-05 | 3.74E-06 | | 5.07E-08 | 5.89E-08 | 2.01E-06 | 1.23E-03 | 7.71E-07 | 2.14E-11 | 1.54E-04 | | 2.73E-10 |
| W_1 (after) (S√s) | 1.36E-05 | 3.98E-05 | | 2.91E-06 | 2.01E-06 | -1.24E-06 | 4.92E-05 | 2.33E-05 | -1.90E-08 | -1.90E-05 | | 6.97E-07 |
| Rs_2 (after) (Ω) | 9.15E-02 | 1.42E+03 | | 9.39E+02 | 3.44E+01 | 9.22E+03 | 2.24E+04 | 1.01E-01 | 1.23E+04 | 5.73E-06 | | 5.26E+00 |
| Cdl_2 (after) (F) | 2.03E-11 | 7.80E-10 | | 3.79E-11 | 1.97E-11 | 8.73E-07 | 3.07E-09 | 2.68E-11 | 3.74E-09 | 2.49E-11 | | 2.11E-11 |
| Rct_2 (after) (Ω) | 6.91E+03 | 4.39E+02 | | 3.18E+03 | 1.18E+05 | 3.44E+04 | 2.80E+03 | 2.70E+00 | 1.56E+03 | 8.79E+00 | | 1.30E+01 |
| W_2 (after) (S√s) | -2.90E-09 | 3.09E-06 | | 2.14E-06 | 9.87E-09 | 3.09E-07 | 1.05E-05 | -1.91E-08 | 2.88E-05 | -1.23E-08 | | -1.85E-08 |
| Rs_3 (after) (Ω) | 1.64E+04 | 9.49E+03 | | 1.18E+04 | 9.96E+03 | 1.56E+00 | 1.14E-02 | 9.63E+03 | 8.59E+03 | 4.77E+04 | | 1.39E+04 |
| Rct_3 (after) (Ω) | 8.82E+00 | 1.05E+02 | | 1.21E+01 | 6.74E+03 | 4.16E+04 | 1.01E+03 | 7.00E+02 | 2.80E+04 | 1.80E-05 | | 6.08E+04 |
| Cdl_3 (after) (F) | 1.76E-07 | 4.46E-10 | | 3.70E-05 | 1.68E-06 | 1.64E-11 | 2.11E-11 | 2.56E-05 | 6.25E-06 | 6.03E-07 | | 5.33E-06 |
| W_3 (after) (S√s) | 7.46E-06 | 8.73E-08 | | -1.27E-04 | 4.40E-06 | 9.52E-07 | -1.26E-08 | 3.50E-05 | 5.95E-05 | 2.23E-06 | | -4.06E-06 |
| Rs_1 (err) (Ω) | 8.70E+04 | 3.29E+02 | | 6.03E+03 | 1.80E+05 | 7.76E+04 | 6.36E+04 | 5.50E+05 | 1.38E+03 | 4.53E+04 | | 4.34E+03 |
| Rct_1 (err) (Ω) | 1.62E+11 | 3.29E+03 | | 1.57E+04 | 2.84E+05 | 4.47E+05 | 8.85E+09 | 5.78E+05 | 2.23E+08 | 6.47E+06 | | 3.22E+03 |
| Cdl_1 (err) (F) | 3.00E+01 | 9.10E-07 | | 1.34E-07 | 1.64E-06 | 9.50E-06 | 8.50E-01 | 3.69E-04 | 8.00E-08 | 1.47E-02 | | 6.88E-11 |
| W_1 (err) (S√s) | 1.18E-04 | 3.32E-05 | | 7.44E-06 | 2.77E-05 | 6.44E-06 | 9.51E+01 | 1.60E-03 | 1.03E-08 | 3.89E-02 | | 1.85E-07 |
| Rs_2 (err) (Ω) | 6.76E+04 | 5.07E+02 | | 7.03E+03 | 8.27E+03 | 6.97E+03 | 1.31E+05 | 7.47E+02 | 3.11E+05 | 3.50E+02 | | 9.25E+02 |
| Cdl_2 (err) (F) | 1.30E-09 | 6.54E-10 | | 1.84E-10 | 7.77E-12 | 5.89E-06 | 3.68E-08 | 1.70E-08 | 1.91E-07 | 2.93E-09 | | 3.17E-09 |
| Rct_2 (err) (Ω) | 1.47E+08 | 8.12E+02 | | 1.23E+04 | 3.07E+05 | 6.87E+07 | 3.52E+04 | 4.65E+07 | 8.47E+04 | 1.92E+07 | | 9.29E+06 |
| W_2 (err) (S√s) | 3.55E-08 | 9.59E-07 | | 9.08E-06 | 3.71E-08 | 1.96E-05 | 1.43E-04 | 6.35E-09 | 1.67E-03 | 1.22E-09 | | 7.90E-09 |
| Rs_3 (err) (Ω) | 8.00E+05 | 1.84E+04 | | 3.55E+03 | 2.25E+04 | 3.11E+02 | 9.18E+02 | 5.22E+05 | 1.53E+05 | 5.78E+05 | | 3.59E+02 |
| Rct_3 (err) (Ω) | 1.09E+07 | 3.22E+07 | | 7.47E+05 | 1.85E+05 | 3.26E+05 | 3.38E+05 | 1.98E+07 | 2.32E+06 | 1.43E+12 | | 8.91E+04 |
| Cdl_3 (err) (F) | 5.99E-04 | 2.45E-07 | | 1.24E-02 | 2.39E-06 | 4.04E-13 | 5.76E-11 | 2.15E-02 | 1.94E-04 | 7.13E+00 | | 6.69E-07 |
| W_3 (err) (S√s) | 5.34E-04 | 1.07E-06 | | 1.36E-03 | 2.07E-05 | 1.51E-05 | 4.96E-09 | 3.69E-03 | 3.02E-03 | 4.79E-05 | | 1.88E-05 |
| Goodness of fit (after) | 1.98E-05 | 4.49E-05 | | 1.11E-04 | 4.82E-06 | 1.36E-03 | 2.52E-03 | 1.34E-03 | 8.40E-04 | 2.51E-03 | | 2.51E-03 |
| Rs_1 (before) (Ω) | 5.97E+04 | 4.26E-02 | | 1.91E+04 | 8.58E-02 | 1.14E+04 | 1.69E-03 | 2.12E+04 | 6.59E+04 | 6.73E-03 | | 5.07E+04 |
| Rct_1 (before) (Ω) | 9.57E+03 | 2.21E+04 | | 9.50E+03 | 4.28E+04 | 8.91E-01 | 1.86E-03 | 9.46E+04 | 7.59E+03 | 6.66E-01 | | 1.54E+04 |
| Cdl_1 (before) (F) | 2.80E-07 | 1.55E-11 | | 4.77E-07 | 1.60E-11 | 8.00E-07 | 2.48E-11 | 9.83E-04 | 5.05E-10 | 2.95E-11 | | 3.25E-10 |
| W_1 (before) (S√s) | 6.61E-06 | 2.04E-06 | | -2.29E-06 | 8.47E-07 | -1.16E-06 | -9.43E-09 | -1.23E-03 | 6.17E-07 | -2.42E-08 | | 1.03E-06 |
| Rs_2 (before) (Ω) | 8.87E+01 | 9.42E+03 | | 5.73E+04 | 1.45E+04 | 7.52E+03 | 9.19E+04 | 6.50E-02 | 1.74E+04 | 4.18E+04 | | 1.98E+04 |
| Cdl_2 (before) (F) | 1.78E-11 | 9.47E-07 | | 1.74E-11 | 7.24E-07 | 2.37E-11 | 7.04E-11 | 2.73E-11 | 2.14E-05 | 6.21E-11 | | 8.55E-06 |
| Rct_2 (before) (Ω) | 1.44E+05 | 1.92E+04 | | 1.63E+04 | 9.77E+03 | 2.25E+04 | 1.35E+05 | 1.47E+00 | 3.34E-05 | 1.20E+05 | | 6.54E+03 |
| W_2 (before) (S√s) | 2.14E-08 | -5.18E-07 | | -1.63E-08 | -7.32E-07 | 3.04E-06 | 8.13E-07 | -2.29E-08 | -9.61E-06 | 6.45E-08 | | 1.51E-04 |
| Rs_3 (before) (Ω) | 1.65E+04 | 1.91E+04 | | 6.71E+03 | 2.53E+04 | 1.93E+04 | 5.36E+04 | 4.37E+04 | 3.84E-06 | 2.27E+04 | | 1.37E+00 |
| Rct_3 (before) (Ω) | 4.43E-01 | 1.14E+05 | | 3.57E+03 | 1.02E+04 | 1.71E+03 | 7.06E-01 | 7.75E+04 | 1.40E+02 | 2.91E+03 | | 1.53E+03 |
| Cdl_3 (before) (F) | 1.08E-05 | 2.23E-07 | | 4.62E-07 | 2.22E-07 | 7.92E-08 | 1.96E-05 | 5.85E-11 | 1.87E-11 | 1.26E-05 | | 1.99E-11 |
| W_3 (before) (S√s) | 1.02E-05 | 8.77E-07 | | 5.74E-06 | 2.47E-06 | 2.82E-06 | -2.06E-06 | 7.03E-08 | -1.17E-08 | 3.06E-04 | | -1.41E-08 |
| Rs_1 (err) (Ω) | 9.90E+07 | 8.74E+03 | | 1.41E+04 | 5.45E+03 | 2.32E+04 | 1.06E+02 | 1.75E+02 | 1.30E+04 | 5.13E+02 | | 1.53E+04 |
| Rct_1 (err) (Ω) | 3.17E+07 | 5.17E+04 | | 3.62E+03 | 2.48E+05 | 3.35E+10 | 3.70E+10 | 2.95E+10 | 8.66E+03 | 9.76E+07 | | 1.42E+04 |
| Cdl_1 (err) (F) | 8.97E-04 | 1.24E-11 | | 2.72E-07 | 4.33E-12 | 4.52E-02 | 3.29E-06 | 3.21E-02 | 7.59E-10 | 5.71E-08 | | 2.94E-10 |
| W_1 (err) (S√s) | 2.40E-02 | 9.54E-06 | | 5.26E-07 | 1.02E-05 | 7.76E-06 | 1.01E-09 | 4.42E+05 | 3.14E-07 | 1.02E-08 | | 1.15E-06 |
| Rs_2 (err) (Ω) | 3.77E+03 | 7.53E+04 | | 1.83E+04 | 7.97E+04 | 1.73E+04 | 7.30E+05 | 6.18E+02 | 7.61E+02 | 1.20E+04 | | 2.73E+03 |
| Cdl_2 (err) (F) | 1.87E-12 | 5.77E-06 | | 3.42E-12 | 3.45E-06 | 1.10E-10 | 1.10E-09 | 2.98E-08 | 5.54E+02 | 1.78E-11 | | 8.13E-06 |
| Rct_2 (err) (Ω) | 8.45E+04 | 8.43E+05 | | 6.04E+03 | 4.29E+05 | 2.54E+05 | 3.67E+06 | 5.69E+07 | 6.00E+12 | 5.88E+04 | | 2.84E+04 |
| W_2 (err) (S√s) | 4.48E-08 | 1.44E-05 | | 2.79E-09 | 7.27E-06 | 6.52E-05 | 2.32E-05 | 1.21E-08 | 1.52E-04 | 1.78E-08 | | 4.85E-03 |
| Rs_3 (err) (Ω) | 7.61E+06 | 3.38E+05 | | 1.76E+03 | 3.21E+05 | 1.78E+05 | 2.46E+05 | 1.67E+04 | 8.99E+02 | 2.45E+02 | | 8.37E+02 |
| Rcl_3 (err) (Ω) | 1.55E+10 | 8.14E+06 | | 6.71E+03 | 4.71E+05 | 4.30E+04 | 2.36E+13 | 3.75E+04 | 2.78E+06 | 7.32E+03 | | 1.18E+05 |
| Cdl_3 (err) (F) | 1.61E+00 | 5.47E-06 | | 1.46E-07 | 2.70E-06 | 1.56E-06 | 1.00E+02 | 2.00E-11 | 3.81E-10 | 7.43E-06 | | 2.71E-11 |
| W_3 (err) (S√s) | 1.36E-02 | 2.05E-05 | | 1.13E-06 | 1.37E-05 | 5.48E-05 | 1.49E-03 | 1.29E-08 | 5.10E-09 | 6.28E-03 | | 4.55E-09 |
| Goodness of fit (before) | 1.19E-05 | 5.51E-05 | | 4.57E-04 | 7.95E-05 | 5.55E-05 | 2.45E-03 | 8.68E-04 | 3.81E-03 | 2.49E-03 | | 4.59E-03 |
| Rs_1 (diff) (Ω) | -5.43E+04 | 1.01E+04 | | -1.54E+04 | 2.84E+04 | 9.34E+03 | 1.56E+04 | -1.13E+04 | -6.59E+04 | 1.34E+04 | | -1.93E+04 |
| Rct _1(diff) (Ω) | -9.57E+03 | -1.82E+04 | | -9.14E+03 | -4.21E+04 | 6.51E+04 | 1.41E+04 | -9.46E+04 | -7.59E+03 | 2.37E+03 | | -2.11E+03 |
| Cdl_1 (diff) (F) | 1.20E-05 | 3.74E-06 | | -4.26E-07 | 5.89E-08 | 1.21E-06 | 1.23E-03 | -9.82E-04 | -4.84E-10 | 1.53E-04 | | -5.21E-11 |
| W_1 (diff) (S√s) | 6.99E-06 | 3.77E-05 | | 5.21E-06 | 1.16E-06 | -7.70E-08 | 4.92E-05 | 1.25E-03 | -6.36E-07 | -1.89E-05 | | -3.32E-07 |
| Rs_2 (diff) (Ω) | -8.86E+01 | -8.00E+03 | | -5.63E+04 | -1.44E+04 | 1.70E+03 | -6.95E+04 | 3.65E-02 | -5.18E+03 | -4.18E+04 | | -1.98E+04 |
| Cdl_2 (diff) (F) | 2.52E-12 | -9.46E-07 | | 2.06E-11 | -7.23E-07 | 8.72E-07 | 2.99E-09 | -4.90E-13 | -2.14E-05 | -3.72E-11 | | -8.55E-06 |
| Rct_2 (diff) (Ω) | -1.37E+05 | -1.87E+04 | | -1.31E+04 | 1.08E+05 | 1.19E+04 | -1.32E+05 | 1.23E+00 | 1.56E+03 | -1.20E+05 | | -6.53E+03 |
| W_2 (diff) (S√s) | -2.43E-08 | 3.61E-06 | | 2.16E-06 | 7.41E-07 | -2.73E-06 | 9.73E-06 | 3.77E-09 | 3.84E-05 | -7.68E-08 | | -1.51E-04 |
| Rs_3 (diff) (Ω) | -1.70E+02 | -9.60E+03 | | 5.09E+03 | -1.53E+04 | -1.93E+04 | -5.36E+04 | -3.41E+04 | 8.58E+03 | 2.51E+04 | | 1.39E+04 |
| Rct_3 (diff) (Ω) | 8.38E+00 | -1.14E+05 | | -3.56E+03 | -3.49E+03 | 3.99E+04 | 1.01E+03 | -7.68E+04 | 2.79E+04 | -2.91E+03 | | 5.93E+04 |
| Cdl_3 (diff) (F) | -1.06E-05 | -2.22E-07 | | 3.65E-05 | 1.46E-06 | -7.92E-08 | -1.96E-05 | 2.56E-05 | 6.24E-06 | -1.20E-05 | | 5.33E-06 |
| W_3 (diff) (S√s) | -2.73E-06 | -7.90E-07 | | -1.32E-04 | 1.94E-06 | -1.86E-06 | 2.05E-06 | 3.49E-05 | 5.95E-05 | -3.04E-04 | | -4.05E-06 |
| R-total (after) (Ω) | 2.87E+04 | 2.53E+04 | | 1.99E+04 | 1.64E+05 | 1.71E+05 | 5.59E+04 | 2.02E+04 | 5.04E+04 | 6.35E+04 | | 1.19E+05 |
| R-total (before) (Ω) | 2.30E+05 | 1.84E+05 | | 1.12E+05 | 1.03E+05 | 6.23E+04 | 1.46E+05 | 2.37E+05 | 9.10E+04 | 6.74E+04 | | 8.74E+04 |
| R-total (diff) (Ω) | -2.01E+05 | -1.58E+05 | | -9.24E+04 | 6.13E+04 | 1.09E+05 | -2.24E+05 | -2.17E+05 | -4.06E+04 | -1.24E+05 | | 2.55E+04 |
| C-total (after) (F) | 3.74E-06 | 3.70E-05 | | 1.74E-06 | 2.88E-06 | 0.00E+00 | 1.23E-03 | 2.64E-05 | 6.25E-06 | 1.54E-04 | | 5.33E-06 |
| C-total (before) (F) | 1.10E-05 | 1.17E-06 | | 9.39E-07 | 9.46E-07 | 8.79E-07 | 1.96E-05 | 9.83E-04 | 2.14E-05 | 1.26E-05 | | 8.55E-06 |
| C-total (diff) (F) | 1.41E-06 | 2.57E-06 | | 3.61E-05 | 7.96E-07 | 2.00E-06 | 1.21E-03 | -9.57E-04 | -1.52E-05 | 1.42E-04 | | -3.22E-06 |
| W-total (after) (S√s) | 2.11E-05 | 4.30E-05 | | -1.22E-04 | 6.42E-06 | 2.29E-08 | 5.97E-05 | 5.82E-05 | 8.83E-05 | -1.67E-05 | | -3.38E-06 |
| W-total (before) (S√s) | 1.68E-05 | 2.40E-06 | | 3.43E-06 | 2.58E-06 | 4.70E-06 | -1.26E-06 | -1.23E-03 | -9.01E-06 | 3.06E-04 | | 1.52E-04 |
| W-total (diff) (S√s) | 4.24E-06 | 4.06E-05 | | -1.25E-04 | 3.84E-06 | -4.67E-06 | 6.10E-05 | 1.29E-03 | 9.73E-05 | -3.23E-04 | | -1.56E-04 |
| Real impedance (before) (kΩ) | 1.28E+01 | 4.89E+00 | | 4.95E+00 | 7.66E+00 | 5.79E+00 | 4.54E+01 | 2.04E+01 | 1.49E+01 | 2.21E+01 | | 1.58E+01 |
| Real impedance (after) (kΩ) | 4.07E+00 | 1.23E+00 | | 1.61E+00 | 7.33E+00 | 5.48E+00 | 9.64E+00 | 4.96E+00 | 5.32E+00 | 1.06E+01 | | 1.10E+01 |
| Real impedance (diff) (kΩ) | -8.70E+00 | -3.66E+00 | | -3.34E+00 | -3.27E-01 | -3.15E-01 | -3.58E+01 | -1.54E+01 | -9.57E+00 | -1.15E+01 | | -4.71E+00 |
| R-total (%diff) (%) | -8.75E+01 | -8.62E+01 | | -8.23E+01 | 5.98E+01 | 1.74E+02 | -1.54E+02 | -9.15E+01 | -4.46E+01 | -1.84E+02 | | 2.92E+01 |
| Real impedance (%diff) (%) | -6.81E+01 | -7.48E+01 | | -6.74E+01 | -4.27E+00 | -5.44E+00 | -7.88E+01 | -7.57E+01 | -6.43E+01 | -5.20E+01 | | -2.99E+01 |

Table S3: The ANOVA table for the derived R-Total (diff) model.

| R-Total (diff) | | | | | | | | | | | | | |
| --- | --- | --- | --- | --- | --- | --- | --- | --- | --- | --- | --- | --- | --- |
| Source | Sum of Squares | | | df | Mean Square | | | F Value | p-value Prob > F | | |  | |
| Model | 1.114E+011 | | | 1 | 1.114E+011 | | | 49.23 | 0.0001 | | | significant | |
| A-Concentration | 1.114E+011 | | | 1 | 1.114E+011 | | | 49.23 | 0.0001 | | |  | |
| Residual | 1.811E+010 | | | 8 | 2.263E+009 | | |  |  | | |  | |
| Cor Total | 1.346E+011 | | | 10 |  | | |  |  | | |  | |
| R^2^ | | 0.8602 | | | | Predicted R^2^ | | | | 0.7767 | | |  |
| Adjusted R^2^ | | | 0.8427 | | | | Adequate Precision | | | | 13.602 | | |

Table S4: The ANOVA table for the derived Real Impedance (%diff) model.

| Real Impedance (%diff) | | | | | | | | | | | | | |
| --- | --- | --- | --- | --- | --- | --- | --- | --- | --- | --- | --- | --- | --- |
| Source | Sum of Squares | | | df | Mean Square | | | F Value | p-value Prob > F | | |  | |
| Model | 5163.90 | | | 1 | 5163.90 | | | 26.29 | 0.0009 | | | significant | |
| A-Concentration | 5163.90 | | | 1 | 5163.90 | | | 26.29 | 0.0009 | | |  | |
| Residual | 1571.11 | | | 8 | 196.39 | | |  |  | | |  | |
| Cor Total | 7424.99 | | | 10 |  | | |  |  | | |  | |
| R^2^ | | 0.7667 | | | | Predicted R^2^ | | | | 0.5870 | | |  |
| Adjusted R^2^ | | | 0.7376 | | | | Adequate Precision | | | | 10.847 | | |

Table S5: A list of estimated costs per sensor and a resulting estimated production cost per sensor. Costs of water, ethanol, and 1,4-dithiobenzene were estimated based on the observation that at least 50 sensors could be prepared for functionalisation in the single, 100mL of reagent used for each step.

| Material | Mass per sensor | Unit | Price ($/unit) | Cost per sensor ($) |
| --- | --- | --- | --- | --- |
| COC | 0.18 | g | 0.03 | 0.01 |
| Gold | 0.02 | g | 63.53 | 1.57 |
| DI Water | 2.5 | g | 0 | 0 |
| Ethanol | 0.78 | g | 0 | 0 |
| 1,4-Dithiobenzene | 0.02 | g | 110 | 1.99 |
| 6-Mercaptohexanol | 0.1 | ml | 12.28 | 1.23 |
| Electricity | 0.07 | Whr | 0.17 | 0.01 |
|  |  |  | Total | 4.78 |

Table S6: The CAP deposition parameters used during sensor fabrication.

| Parameter | Value |
| --- | --- |
| Fluence | 0.79 J/cm^2^ |
| Wavelength | 1064 nm |
| Spot Size | 140 µm |
| Spot Profile | Gaussian |
| Pulse Repetition Frequency | 10 kHz |
| Pulse Width | 3.5 µs |
| Scan Speed | 18 mm/s |
| Scan Strategy | Bidirectional Raster |
| Vertical Spacing | 50 µm |


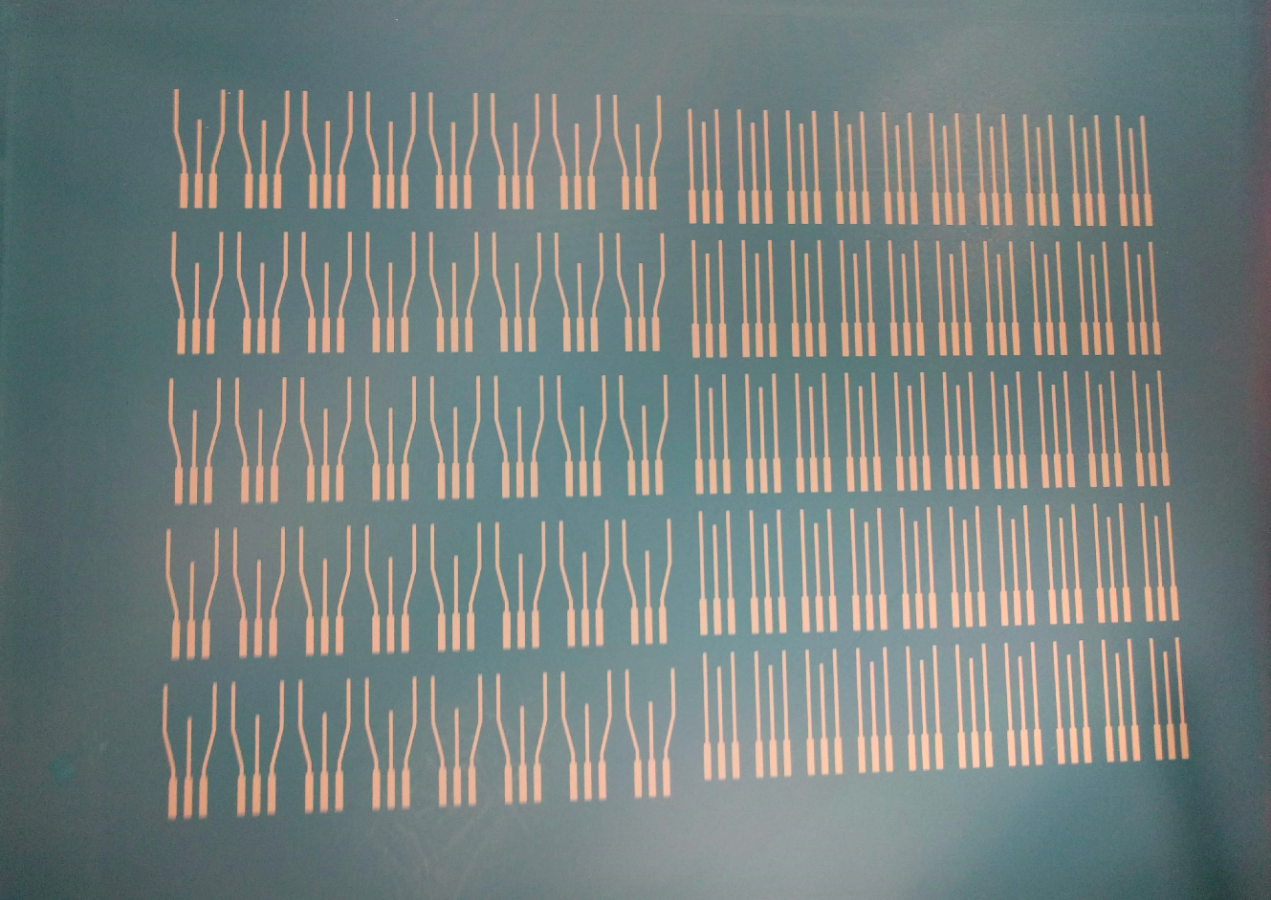
Fig. S10: A photograph of the screen used for the screen printing step of the fabrication process. The template on the right half of the screen was used for the experimental work presented here.

Table S7: The LASiS parameters used during sensor fabrication.

| Parameter | Value |
| --- | --- |
| Fluence | 0.79 J/cm^2^ |
| Wavelength | 1064 nm |
| Spot Size | 140 µm |
| Spot Profile | Gaussian |
| Pulse Repetition Frequency | 10 kHz |
| Pulse Width | 700 ps |
| Scan Speed | 2 mm/s |
| Scan Strategy | Spiral |
| Flow Rate | 140 mL/min |
| Production Time | 30 mins |


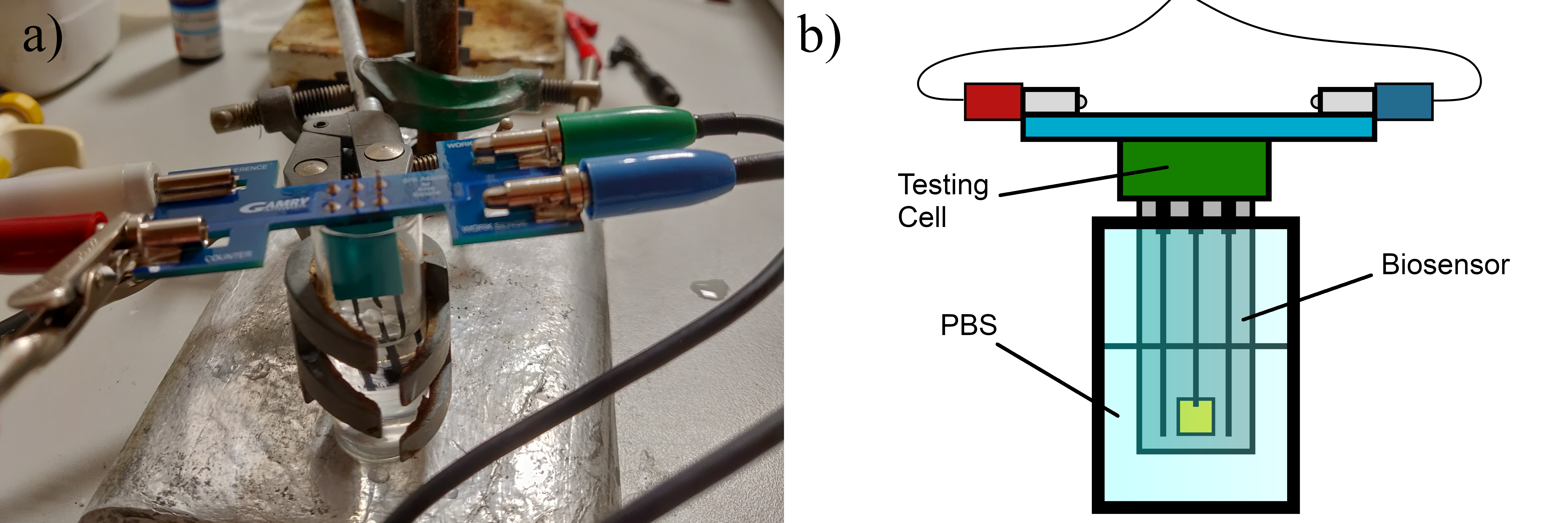
Fig. S11: a) A photograph and b) schematic of the electrochemical testing setup used during the course of this experimental study.

Table S8: The EIS parameters used during electrochemical testing.

| Parameter | Value |
| --- | --- |
| DC Voltage | 0 V vs. Eref |
| AC Voltage | 10 mV |
| Initial Frequency | 1000000Hz |
| Final Frequency | 10 Hz |
| Points/Decade | 100 |
| Area | 0.25 cm^2^ |
| Initial Delay | Off |
